# Analysis of gene expression changes associated with human carcinoma-associated fibroblasts in non-small cell lung carcinoma

**DOI:** 10.1186/s40659-017-0108-9

**Published:** 2017-02-23

**Authors:** Xiaofen Wu, Lei Ruan, Yi Yang, Qi Mei

**Affiliations:** 1 Department of Gerontology, Tongji Hospital, Tongji Medical College, Huazhong University of Science and Technology, Wuhan, 430030 China; 2 Department of Oncology, Tongji Hospital, Tongji Medical College, Huazhong University of Science and Technology, Jiefang Avenue 1095, Wuhan, 430030 China

**Keywords:** Carcinoma-associated fibroblast, Non-small cell lung carcinoma, Differentially expressed genes, Allele-specific copy number, Functional analysis

## Abstract

**Background:**

This study aimed to investigate the gene expression changes associated with carcinoma-associated fibroblasts (CAFs) involving in non-small cell lung carcinoma (NSCLC).

**Methods:**

We downloaded the GEO series GSE22862, which contained matched gene expression values for 15 CAF and normal fibroblasts samples, and series GSE27289 containing SNP genotyping for four matched NSCLC samples. The differentially expressed genes in CAF samples were identified using the limma package in R. Then we performed gene ontology (GO) and pathway enrichment analysis and protein–protein interaction (PPI) network construction using the identified DEGs. Moreover, aberrant cell fraction, ploidy, allele-specific copy number, and loss of heterozygosity (LOH) within CAF cells were analyzed using the allele-specific copy number analysis.

**Results:**

We obtained 545 differentially expressed genes between CAF and normal fibroblasts samples. The up-regulated genes are mainly involved in GO terms such as positive regulation of cell migration and extracellular region, while the down-regulated genes participate in the lung development and extracellular region. Multiple genes including bone morphogenetic protein 4 (*BMP4*) and transforming growth factor, beta 3 (*TGFB3*) are involved in the TGF-β signaling pathway. Genes including *BMP4*, *TGFBI* and matrix Gla protein (*MGP*) were hub genes. Moreover, no LOH event for *BMP4* and *MGP* was found, that for sphingosine kinase 1 (*SPHK1*) was 70%, and for *TGFBI* was 40%.

**Conclusion:**

Our data suggested that *BMP4*, *MGP*, *TGFBI*, and *SPHK1* may be important in CAFs-associated NSCLC, and the abnormal expression and high LOH frequency of them may be used as the diagnosis targets of CAFs in NSCLC.

## Background

Lung cancer is one of the most common leading cause of cancer deaths worldwide [1]. Statistics show that the non-small cell lung carcinoma (NSCLC) accounts for about 85% of all lung cancers in the world [2]. Treatment methods such as surgery, drug therapy and chemotherapy have played certain roles in curing NSCLC [2]. However, in [3] and [4], the authors suggest that 5-year survival rate is poor due to the difficulties in the early diagnose of NSCLC and the easy invasion and metastasis of NSCLC cells. Therefore, exploring the mechanism in NSCLC metastasis and invasion will be of great significance to provide basis for NSCLC diagnosis and treatment.

In [5], authors suggest that carcinoma-associated fibroblasts (CAFs) are the most important component of developing cancers. Recent studies implied that CAFs played important roles in cancers biology occurring in epithelia, such as neoplastic progression, tumor growth, invasion and metastasis [6–8]. CAFs constitute a major portion of the reactive tumor stroma and are related to the cell invasion and metastasis in NSCLC during malignancy [9]. Also, Neta et al. [10] referred that CAFs activities promoted the macrophage recruitment, neovascularization, and tumor growth in incipient neoplasia to orchestrate tumor inflammation via the NF-kB signaling pathway. Furthermore, an increasing number of evidences show that mutations of genes for CAFs are crucial for cancer metastasis and growth. For examples, some changes in DNA copy number of cluster CAFs may contribute to the metastasis for breast cancer cells [11]. In spite of many researches devoted to the exploration of the role of CAFs in NSCLC, the mechanism of CAFs in NSCLC still remains not fully understood.

Using the GEO (Gene Expression Omnibus) series GSE22862, Horie et al. [12] proved that RNAi-mediated targeting of transforming growth factor, beta 1 (TGFB1) ligands were beneficial for lung cancer treatment through its action on cancer and stromal cells. In this study, we screened the differentially expressed genes (DEGs) in NSCLC samples compared with the normal samples using the same GEO series GSE22862 [13]. Bioinformatics methods were used to analyze the functions and pathways of the DEGs, as well as the allele-specific copy number (ASCN) of them to predict hub genes which were related with the CAFs in NSCLC. This study aimed to explore the underlying genes associated with pathomechanism of CAFs in NSCLC, which may help to search for diagnosis and treatment targets for this disease.

## Methods

### Microarray data

We downloaded the GEO series GSE22874 [13] from the GEO database in NCBI (http://www.ncbi.nlm.nih.gov/geo/). This series contains four subseries and two of them (GSE22862 and GSE27289) were analyzed in this study. The single nucleotide polymorphism (SNP) series GSE27289 is generated from four paired primary NSCLC CAF and normal fibroblasts (NF) samples based on the platform of GPL13135 HumanOmniExpress BeadChip. GSE22862 is a CAF series, which originates from matched gene expression values from 15 CAF and NF samples of resected NSCLC tissues based on the platform of GPL5175 Affymetrix Human Exon 1.0 ST Array (Affymetrix Inc., Santa Clara, California, USA). At present, we chose four matched samples (4 CAF samples and 4 NF samples) of two stages I squamous cell carcinomas and 2 stage II adenocarcinoma from the 30 samples of GSE28682.

### Microarray data preprocessing and conversion

The CEL file data of GSE22862 download from the GEO database have been normalized using the Robust Multi-array Analysis method [14] in affy (http://www.bioconductor.org/packages/release/bioc/html/affy.html) package in Bioconductor. When multiple probes correspond to the same gene, the mean expression value was calculated and considered as the expression value of this gene. Meanwhile, the CEL file data of GSE27289 that have been preprocessed using the GenomeStudio [15], were extracted for the SNP symbols, chromosome number, chromosome location, B allele frequency and Log R values both in CAF samples and in NF samples.

### Screening and functional enrichment analysis of DEGs

We screened the DEGs between CAF and NF samples in GSE22862 series using the limma package in R (http://www.bioconductor.org). The p value <0.05 and |log_2_ (fold change)| ≥0.58 (fold change ≥1.5 or ≤0.67) were chosen as the thresholds. In the present study, we did not performed multiple test correction for the p values, because the corrected p values were too low to select enough significant genes.

In addition, we conducted gene ontology (GO) and Kyoto Encyclopedia of Genes and Genomes (KEGG) pathways enrichment analyses for the DEGs using the Database for Annotation Visualization and Integrated Discovery (DAVID, http://david.abcc.ncifcrf.gov/) [16] online tool. p value <0.05 was chosen as threshold.

### Protein–protein interaction (PPI) network construction of DEGs

PPI analysis can provide new insights into protein function, besides, it may help to uncover the generic organization principles of functional cellular networks [17], therefore, we would construct PPI network to further analyze the DEGs. Search Tool for the Retrieval of Interacting Genes/Proteins (STRING, http://string.embl.de/) [18] that provided functional associations between proteins was used to predict the interaction pairs of the selected DEGs. In this study, we only mapped the DEGs into STRING database to predict the PPI pairs because we intended to investigate the interactions between DEGs. Cytoscape (http://www.cytoscape.org/) [19] that was widely used to integrate biomolecular interaction networks into models was used to construct the PPI network of the DEGs. Most of previous obtained biological networks were found to obey the scale-free attribution [20]. Thus, we analyzed the connectivity degree of nodes in the PPI network by topological analysis to obtain the important nodes with higher degrees (hub proteins) [21]. Genome-wide studies have shown that deletion of a hub protein is more likely to be lethal than deletion of a non-hub protein, thus, we think that the hub nodes may play important roles in the CAF of NSCLC.

### ASCN analysis

ASCN analysis of tumor [22, 23] is a method that can be used to analyze the aberrant cell fraction, ploidy, ASCN, and loss of heterozygosity (LOH) of tumor cells. The ASCN for one gene was calculated based on the Log R and B allele frequency values of allele B. In this study, we used ASCN method to determine the values of the aberrant cell fraction, ploidy, ASCN and LOH of the selected genes. Twenty-two autosomes plus two gender associated chromosomes were chosen as parameters of the number of the chromosomes.

### Candidate gene identification

On account of GO and KEGG pathway enrichment results, as well as PPI network of the DEGs, we identified the potential critical genes combining with the results of ASCN analysis. Additionally, based on the databases of tumor suppressor (TS) gene [9] and tumor-associated gene (TAG) [10], we identified the DEGs that function as transcription factors, tumor suppressors or oncogenes. Finally, the ASCN values of these identified genes were analyzed.

## Results

### Identified DEGs

With p value <0.05 and |log_2_ (fold change)| ≥0.58, we identified 545 DEGs including 66 up-regulated and 479 down-regulated DEGs in CAF samples compared with NF samples in GSE22862. The number of down-regulated DEGs was far more than that of up-regulated DEGs, suggesting the important roles of down-regulated genes in NSCLC.

### Functional enrichment analysis of DEGs

The GO functions and pathways of up- and down-regulated DEGs in CAF samples were shown in Tables 1, 2, respectively. Up-regulated genes are significantly involved in GO terms such as cell surface receptor linked signal transduction, G-protein couple receptor protein, positive regulation of cell migration and extracellular region, while down-regulated genes are associated with GO terms including lung development, respiratory tube development and extracellular region (Table 1).

**Table 1:** The enriched gene ontology terms and pathways of the differentially expressed genes (DEGs) in carcinoma-associated fibroblasts (CAF) of non-small cell lung carcinoma (NSCLC)

Category	Term	Count	p value
Up-regulated genes			
BP	GO:0007166~cell surface receptor linked signal transduction	16	4.95E−04
	GO:0007186~G-protein coupled receptor protein signaling pathway	12	7.29E−04
	GO:0008544~epidermis development	5	0.0029
	GO:0030335~positive regulation of cell migration	4	0.0030
	GO:0007398~ectoderm development	5	0.0039
	GO:0040017~positive regulation of locomotion	4	0.0039
	GO:0051272~positive regulation of cell motion	4	0.0039
CC	GO:0005576~extracellular region	13	0.0249
	GO:0005886~plasma membrane	19	0.0456
MF	GO:0005179~hormone activity	3	0.0496
Down-regulated genes			
BP	GO:0007416~synaptogenesis	9	1.31E−06
	GO:0016339~calcium-dependent cell–cell adhesion	8	1.43E−06
	GO:0043062~extracellular structure organization	16	2.72E−05
	GO:0016485~protein processing	12	1.80E−04
	GO:0050808~synapse organization	9	1.89E−04
	GO:0030324~lung development	11	2.80E−04
	GO:0030323~respiratory tube development	11	3.57E−04
CC	GO:0005576~extracellular region	87	2.38E−06
	GO:0044421~extracellular region part	45	2.47E−04
	GO:0005624~membrane fraction	38	8.21E−04
MF	GO:0005509~calcium ion binding	47	2.44E−05
	GO:0015923~mannosidase activity	4	0.0067
	GO:0019838~growth factor binding	9	0.0070

*BP* biological process, *CC* cellular component, *MF* molecular function, *Count* number of genes

**Table 2:** The enriched pathways of the differentially expressed genes (DEGs) in carcinoma-associated fibroblasts (CAF) of non-small cell lung carcinoma (NSCLC)

DEGs	KEGG pathway	Count	p value
Up-regulated gene	hsa04740:olfactory transduction	5	0.0269
Down-regulated gene	hsa04610:complement and coagulation cascades	9	7.57E−04
	hsa05410:hypertrophic cardiomyopathy (HCM)	8	0.0120
	hsa00640:propanoate metabolism	5	0.0128
	hsa05414:dilated cardiomyopathy	8	0.0165
	hsa05412:arrhythmogenic right ventricular cardiomyopathy (ARVC)	7	0.0219
	hsa00380:tryptophan metabolism	5	0.0274
	hsa04350:TGF-beta signaling pathway	7	0.0392
	hsa00340:histidine metabolism	4	0.0499

*Count* number of genes

In addition, only pathway of Olfactory transduction is enriched by up-regulated genes, such as olfactory receptor, family 6, subfamily B, member 1 (*OR6B1*), olfactory receptor, family 5, subfamily L, member 2 (*OR5L2*) and olfactory receptor, family 10, subfamily H, member 1 (*OR10H1*). The down-regulated genes, such as bone morphogenetic protein 4 (*BMP4*), *TGFB3* and SMAD family member 3 (*SMAD3*), are mainly involved in TGF-β signaling pathway (Table 2).

### PPI network construction

**Fig. 1:**
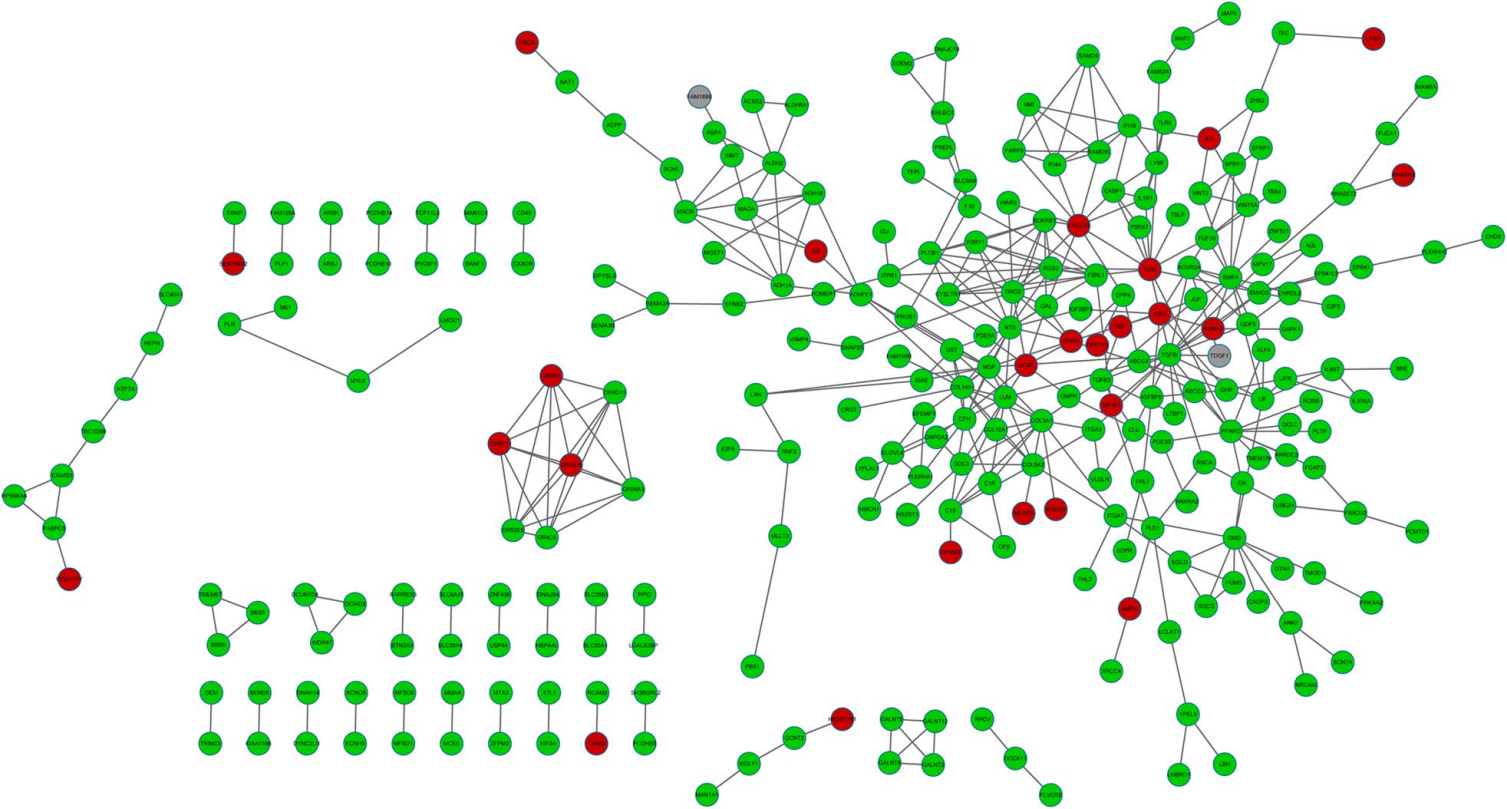
Protein–protein interaction network of the selected differentially expressed genes (DEGs). *Red circle* stands for the up-regulated genes while *green circle* stands for the down-regulated genes. *Gray circle* stands for the non-differentially expressed genes. *Edge* stands for the interaction between two genes

The PPI network of DEGs was constructed and shown in Fig. 1. The PPI network contained 266 nodes and 409 interaction pairs. Among the top ten high degree genes, only two were up-regulated, including toll-like receptor 4 (*TLR4*, degree = 12) and chemokine (C-X-C Motif) ligand 10 (*CXCL10*, degree = 11). The other eight down-regulated genes including transforming growth factor, beta-induced (*TGFBI*, degree = 19), neurotensin (*NTS*, degree = 15), matrix Gla Protein (*MGP*, degree = 13), guanine nucleotide binding protein (G Protein), gamma 2 (*GNG2*, degree = 13), collagen, type III, alpha 1 (*COL3A1,* degree = 13), *BMP4* (degree = 12), peroxisome proliferator-activated receptor (*PPARG*, degree = 12), and collagen, type XIV, alpha 1 (*COL14A1*, degree = 11).

### Analysis of copy number of alleles

The aberrant cell fraction and ploidy of genes of tumor cells in CAF samples were respectively 77% and 2.002 based on the ASCNT analysis. In addition, based on the functional enrichment analysis and PPI network, we obtained 16 critical genes. ASCN results of the 16 critical genes were shown in Table 3. The copy number of alleles of most genes were abnormal except for *MGP*, *BMP4*, *PPARG* and *TLR4*, moreover, they were all positive for LOH. For instance, the LOH proportion of *TGFB3* and sphingosine kinase 1 (*SPHK1*) was 70%, while that of *MGP* was 0% (Table 3).

**Table 3:** Copy numbers of allele and loss of heterozygosity (LOH) for the genes using the allele-specific copy number (ASCN) analysis

Gene symbol	The copy number of A allele	The copy number of B allele	Proportion (%)	Description	Log_2_ FC
TGFB3	1.7	0.3	70	Copy neutral LOH in 70%	−0.96
SPHK1	0.3	1.7	70	Copy neutral LOH in 70%	0.6825
NTS	0.5	1.5	50	Copy neutral LOH in 50%	−0.7975
CXCL10	1.5	0.5	50	Copy neutral LOH in 50%	0.66
F2RL1	0.5	1.5	50	Copy neutral LOH in 50%	−1.375
TGFBI	0.6	1.4	40	Loss of one allele in 40%	−1.23
COL3A1	1.4	0.6	40	Loss of one allele in 50%	−1.12
LUM	1.2	0.8	20	Loss of one allele in 20%	−0.665
IGF2	0.8	1.2	20	Loss of one allele in 20%	1.07125
GNG2	0.9	1.1	10	Imbalance in 10%	−1.3775
COL14A1	1.1	0.9	10	Imbalance in 10%	−1.8125
WNT2	1.1	1	5	Imbalance in 5%	−1.1275
MGP	1	1	0	Expected balance	−2.3075
BMP4	1	1	0	Expected balance	−1.115
PPARG	1	1	0	Expected balance	−1.1
TLR4	1	1	0	Expected balance	1.09

*Gene symbol* the name of one gene, *proportion* the percentage of copy number of A allele minus copy number of B allele, *Description* the frequency of LOH, *FC* fold change

### Identified candidate genes

The result of function annotation for DEGs in CAF samples was shown in Table 4. The results displayed that six up-regulated genes and ten down-regulated genes function as transcription factors. In addition, *SPHK1* and pre-B-cell leukemia homeobox 1 (*PBX1*), etc. were oncogenes, while runt-related transcription factor 3 (*RUNX3*) and *TGFBI*, etc. were tumor suppressor genes (Table 4).

**Table 4:** The allele-specific copy number (ASCN) of the 11 genes with high loss of heterozygosity (LOH) frequency

Gene symbol	The copy number of A allele	The copy number of B allele	Proportion (%)	Description	Log_2_ FC
LXN	2	0	100	Loss of the one allele,gain of another allele	−1.9225
RASL11A	2	0	100	Loss of the one allele,gain of another allele	−0.885
KCNRG	0	2	100	Loss of the one allele,gain of another allele	−0.6275
TFAP2C	0.3	1.7	70	Copy neutral LOH in 70%	0.9875
SPHK1	0.3	1.7	70	Copy neutral LOH in 70%	0.6825
TP53INP1	1.6	0.4	60	Copy neutral LOH in 60%	−0.795
HOXD12	1.5	0.5	50	Copy neutral LOH in 50%	0.87
ERRFI1	1.5	0.5	50	Copy neutral LOH in 50%	−1.195
RPS6KA6	0.6	1.6	50	Loss of one allele in 50%	−1.12
DNAJB4	0.4	1.4	50	Copy neutral LOH in 50%	−1.02
ITGA7	0.5	1.5	50	Copy neutral LOH in 50%	−0.925

*Gene symbol* the name of one gene, *proportion* the percentage of copy number of A allele minus copy number of B allele, *Description* the frequency of LOH, *FC* fold change

The result of ASCN analysis of genes mentioned above was shown in Table 5. As shown in Table 5, LOH of latexin (*LXN*), RAS-like, family 11, member A (*RASL11A*) and potassium channel regulator (*KCNRG*) were all 100%, LOH of transcription factor ap-2 gamma (*TFAP2C*) and *SPHK1* was 70%, of tumor protein P53 inducible nuclear protein 1 (*TP53INP1*) was 60%, and of homeobox D12 (*HOXD12*), ERBB receptor feedback inhibitor 1 (*ERRFI1*), ribosomal protein S6 kinase, 90 kDa, polypeptide 6 (*RPS6KA6*), DnaJ (Hsp40) homolog, subfamily B, member 4 (*DNAJB4*) and integrin, alpha 7 (*ITGA7*) was 50%. Moreover, the copy numbers of allele for these genes were not the same (Table 5).

**Table 5:** Function annotation of differentially expressed genes (DEGs) in carcinoma-associated fibroblasts (CAF)

	TF genes	Oncogenes	TSG
Up-regualted	RUNX3, MEOX2, FOXE1, HOXD12, TFAP2C, ID1	SPHK1	RUNX3
Down-regulated	TBX4, RORB, PPARG, KLF4, IFI16, PBX1, SOX5, NFIA, SMAD3, FOXP2	TEC, MLF1, PBX1, BANF, MLLT3,CDON	LXN, DPP4, CLU, RARRES1, TGFBI, IGFBP5, ERRFI1, CADM1, JUP, DIRAS3, TXNIP, RARRES3, RPS6KA6, DNAJB4, WNT5A, SFRP1, ITGA7, DAPK1, RASL11A, EPB41, TP53INP1, SCARA3, HBP1, LIMD1, FBXO32, SMAD3, RNASET2, KCNRG, SEMA3B, NRCAM

*TF* transcription factor, *TSG* tumor suppressor gene

## Discussion

In this study, we screened a total of 66 up-regulated and 479 down-regulated DEGs by comparing gene expression between CAFs tissues and NF tissues in NSCLC. DEGs including *BMP4*, *TGFB3* and *MGP* involving in the TGF-β signaling pathway were found to be hub genes in the PPI network. Moreover, no LOH was found in both *BMP4* and *MGP*. Besides, LOH of the tumor oncogene *SPHK1* was 70%, and of tumor suppressor gene *TGFBI* was 40%.

BMP4 is a member of the bone morphogenetic protein family which belongs to the transforming growth factor (TGF-β) superfamily [24], while no LOH events were found about it. In addition, it was found that *BMP4* was a hub gene in the PPI network and involved in lung development and the TGF-β signaling pathway. Meiou et al. [25] reported that the TGF-β factor signaling which was regulated by the transcriptional co-activator p/CAF (a histone acetyltransferase), played key roles in breast cancer cell migration and invasion. In previous studies, BMP4 was found play important roles in developmental and many cellular processes including invasion and migration of various cancer cells [26, 27]. Since BMP2 is closely associated with NSCLC metastasis [28], BMP4 may be a contributor to affect the behavior and function of CAFs in NSCLC via TGF-β signaling pathway. Meanwhile, TGFB3 as another member of the TGF-β family is also enriched in lung development and TGF-β signaling pathway. It was detected with a high frequency of LOH (70%). Some studies indicated the abnormal expression of TGFB3 in NSCLC [29, 30]. Therefore, abnormal expression of BMP4 and TGFB3, as well the high LOH frequency of TGFB3 may be used as biological indicators for malignant CAFs in NSCLC.

Our data also showed that tumor suppressor TGFBI was a hub protein in the PPI network and LOH value of it was 40%. TGFBI has been reported to suppress tumor cell growth in NSCLC [31] and other types of human lung cancers [32]. Therefore, TGFBI may be a tumor suppressor for NSCLC. Besides, Muraoka et al. [33] proved that blocking TGFBI expression enhanced inhibition of tumor cell migration and metastasis. Also, Levy et al. [34] proved that LOH of TGFBI located on chromosome 19q13.1 contributed to the metastasis of breast cancer cells. Thus, LOH may lead to the down-regulation of TGFBI in this study. On the other hand, Fong et al. [35] said that TGFBI promoted the migration of lung cancer cells. CAFs are important for breast cancer cell migration and metastasis [36]. Based on the previous evidences, we speculated that TGFBI may be a tumor suppressor for NSCLC and may be responsible for the CAFs migration in NSCLC.

SPHK1 catalyzes the phosphorylation of sphingosine to form sphingosine-1-phosphate (S1P), a lipid mediator with both intra- and extracellular functions [37]. Presneau and his colleagues proved that the LOH of SPHK1 assigned to chromosome region at 17q25.1-q25.2, suggesting its key role in regulating early events during the development of sensory ganglia in ovarian cancer [38]. Thus, the higher LOH value may be related to the abnormal expression of SPHK1 in NSCLC samples. Therefore, the positive result of SPHK1 at LOH analysis was consistent with the up-regulation of it. In addition, GO analysis showed that SPHK1 enriched in multiple cancer-related processes including positive regulation of cell migration. Chen et al. [39] reported that the tumor oncogene *SPHK1* which was up-regulated was responsible for tumor cell migration in hepatocellular carcinoma. Moreover, SPHK1 was found to enhance the NSCLC cell apoptosis via activating PI3K/Akt pathway [40]. Besides, it can induce breast cancer cell migration [41]. Therefore, we speculated that over-expressed SPHK1 may be associated with CAFs migration in NSCLC.

Interestingly, some of our results were in accordance with the original publication by Navab et al. [13]. In their study, microarray gene-expression analysis of the 15 matched CAF and NF cell lines identifies 46 differentially expressed genes which are significantly enriched for extracellular proteins regulated by the TGF-β signaling pathway. Our study also found that *BMP4* and *TGFB3* are involved in TGF-β signaling pathway, suggesting the important role of this pathway in CAF-associated NSCLC. Additionally, their study revealed prominent involvement of the focal adhesion and MAPK signaling pathways, which was not identified in our study. This difference may be due to different sample size and different screening threshold for DEGs.

In conclusion, genes such as *BMP4*, *TGFB3*, *TGFBI*, and *SPHK1* may play important roles in CAFs of NSCLC. BMP4 and TGFB3 may be contributor to make effects on CAFs in NSCLC via TGF-β signaling pathway. SPHK1 promoted while TGFBI inhibited the NSCLC progression, and both of them may be associated with CAFs migration in NSCLC. In addition, high frequency of TGFB3, TGFBI and SPHK1 may be useful for distinguishing the normal fibroblasts from malignant CAFs in NSCLC. However, further experimental studies are still needed to confirm our results.

## Abbreviations

ASCN: allele-specific copy number
BMP4: bone morphogenetic protein 4
CAFs: carcinoma-associated fibroblasts
DEGs: differentially expressed genes
GO: gene ontology
LOH: loss of heterozygosity
MGP: matrix Gla protein
NSCLC: non-small cell lung carcinoma
PPI: protein–protein interaction
RUNX3: runt-related transcription factor 3
SPHK1: sphingosine kinase 1
TGF-β: transforming growth factor
TGFB3: transforming growth factor, beta 3
TP53INP1: tumor protein P53 inducible nuclear protein 1
TS: tumor suppressor
